# Supplementary material for: The inner membrane complex protein, IMC55, is dispensable for intraerythrocytic development of Plasmodium falciparum
Source: Microbiol Spectr. 2026 Jun 10;14(7):e03656-25. doi: 10.1128/spectrum.03656-25 (PMC13339899; doi:10.1128/spectrum.03656-25)
Supplement: Supplemental material — Table S2; Fig. S1 to S6. [file spectrum.03656-25-s0001.docx]

**Supplemental Data**

**Supplemental table 1. Raw data from PfERC and control IP.**

**Supplemental table 2. List of primers used to generate IMC55 conditional parasites in this study.**

| **Amplicon** | **Primer** | **Sequence (5'-3')** |
| --- | --- | --- |
| IMC55^apt^ integration Cterm | P1 | CGGATTTTTAGCTGGATTAATTGGATCCC |
| IMC55^apt^ Cterm Fwd | P2 | CCCTATGATATCGTCCACCTGGATATCTAGCCGGATTTCTACTCACC |
| IMC55^apt^ Cterm Rev | P3 | GGTACGTCATAAGGGTATCCGGAGACGTCAAAAATATATGTATTTTCAATTCTTGG |
| IMC55^apt^ 3UTR Fwd | P4 | GCCCCTTTCCGGGCGCGCCGTTATGATCTAAAAAATTTATATTTTCATTAGGAAAATAG |
| IMC55^apt^ 3UTR Rev | P5 | CCGGCTAGATATCCAGGTGGACGATATCATAGGGAAATGTTTTAAAAG |
| Aptamer Integration Rev | P6 | CTAGACTAGGTTCCAAGATCTCCC |
| IMC55^apt^ gRNA | P7 | TAATTCAGAATACATAAAAT |
| IMC55^KO^ Integration 5UTR | P8 | GTATTACAATTTAATTATGTAACAATATTATATATCATTTCACTAATTATGGTTGCCCTTTGCATATTCACTG |
| IMC55^KO^ Integration recodonized Cterm Rev | P9 | GTCTCCTATGTGGGTGTCACTCAATATACTACGTGTCCTAC |
| IMC55^KO^ Cterm Fwd | P10 | CTTCGTATAGCATACATTATACGAAGTTATTATATATGTATATATATATATATTTATATATTTTATATTCTTTTAGATGGAGAATTTTCACCTTCATTC |
| IMC55^KO^ Cterm Rev | P11 | CCGGTACATCATATGGGTAGAATTCAAAAATATAAGTGTTTTCAATTCTTGGC |
| IMC55^KO^ 5UTR Fwd | P12 | GCTATACGAAGTTATTGTATATTATTTTTTTTATTTACTTTGCTATAATTTATATTAAAAAAAAAAAAAAAAAAAAGAAGTAAGTAAGTAAGTAAGTAAGTAAGC |
| IMC55^KO^ 5UTR Rev | P13 | GCTATACGAAGTTATTGTATATTATTTTTTTTATTTACTTTGCTATAATTTATATTAAAAAAAAAAAAAAAAAAAAGAAGTAAGTAAGTAAGTAAGTAAGTAAGC |
| IMC55^KO^ 3UTR Fwd | P14 | GGATGAATTATACAAGTAATAAAGCGCTGTTATGATCTAAAAAATTTATATTTTCATTAGG |
| IMC55^KO^ 3UTR Rev | P15 | GATCTGGATCAGCGCTGGAAAAACGTGTAAAAAAAAAAAAACTAAAATAATTAGAATAATATAG |
| IMC55^KO^ gRNA | P16 | AGCACTGTGAGCTTACTCTT |

**
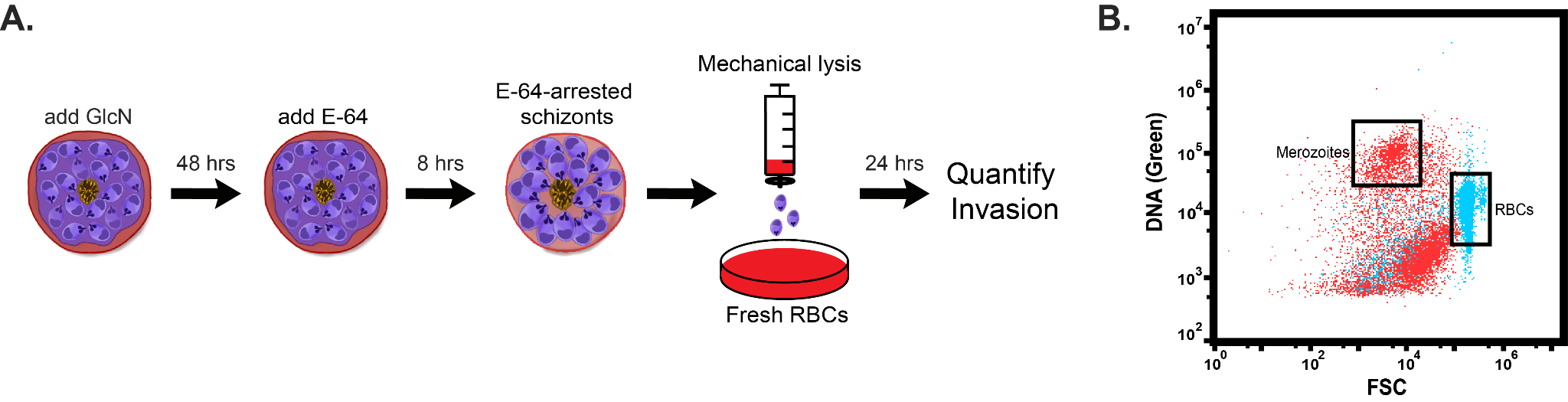
**

**Supplemental Figure 1. PfERC invasion assay.** (A) Schematic showing isolation of infectious merozoites from RBCs and invasion assay. PfERC knockdown was E-64 treated for 8 hours, then parasites were mechanically lysed from host cells, and allowed to invade fresh RBCs. (B) Representative flow cytometry plot showing gating strategy to quantify merozoite (red) and RBC (blue) populations.

**
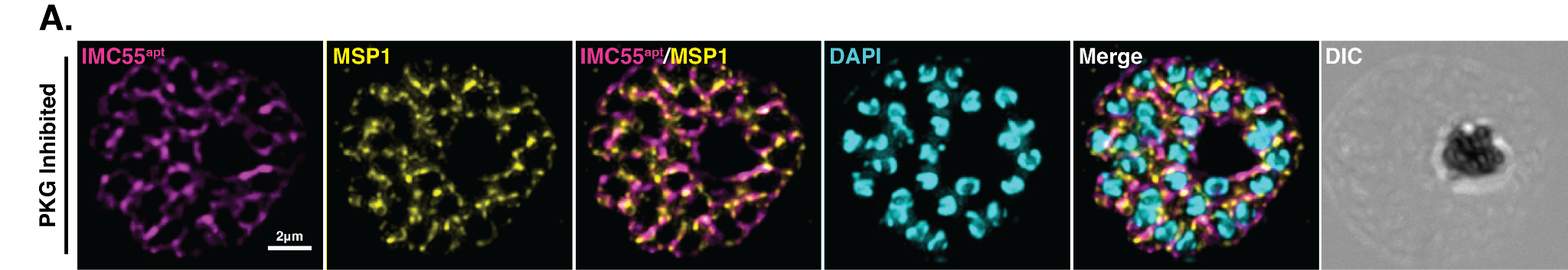
**

**Supplemental Figure 2. IMC55 localization under PKG inhibition.** (A) Representative images from immunofluorescence assay showing subcellular localization of IMC55^apt^ late schizonts stalled using the PKG inhibitor C1 for 4 hours, then fixed via acetone and stained with specific antibodies. Antibodies used were anti-HA to detect IMC55^apt^ (magenta), a marker for the merozoite surface MSP1 (yellow), and the nuclear stain DAPI (cyan). Images include merged fluorescence and DIC. Images were acquired as a Z-stack, deconvolved, then projected as a single image. Representative images from 2 biological replicates.

**
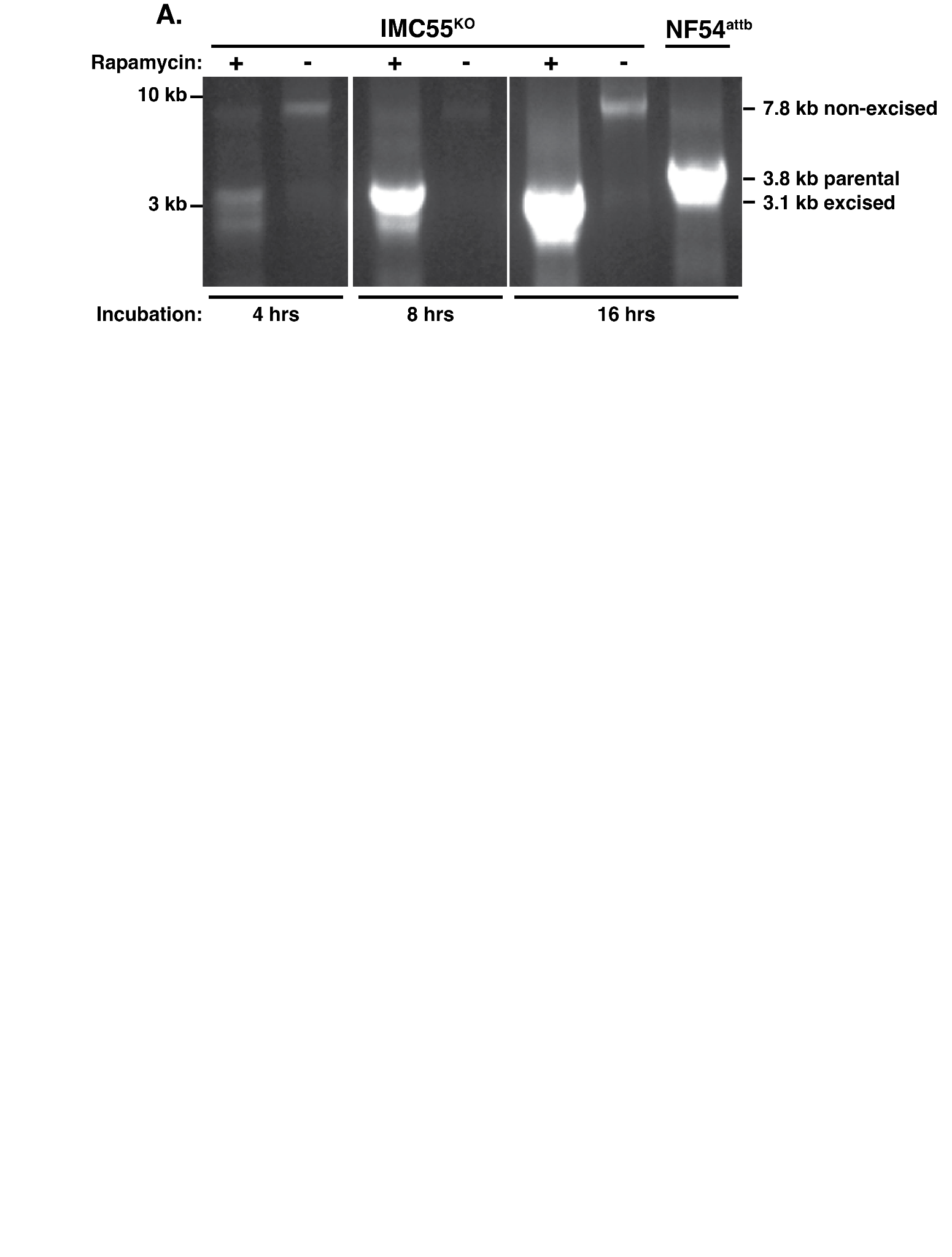
**

**Supplemental Figure 3. Optimization of rapamycin-mediated excision of IMC55^KO^.** (A) PCR verification of IMC55^KO^ excision after incubation with 100 nM rapamycin or 0.05% DMSO for 4, 8, or 16 hours, collected immediately after RAP treatment. Amplification with primer pairs P8 & P15 generates a rapamycin-induced floxed IMC55^KO^ amplicon of 3.1kb, while treatment with DMSO produces a 7.8kb band amplicon. Parental NF54::DiCre amplification generates a 3.8kb amplicon. Representative data from 3 biological replicates.


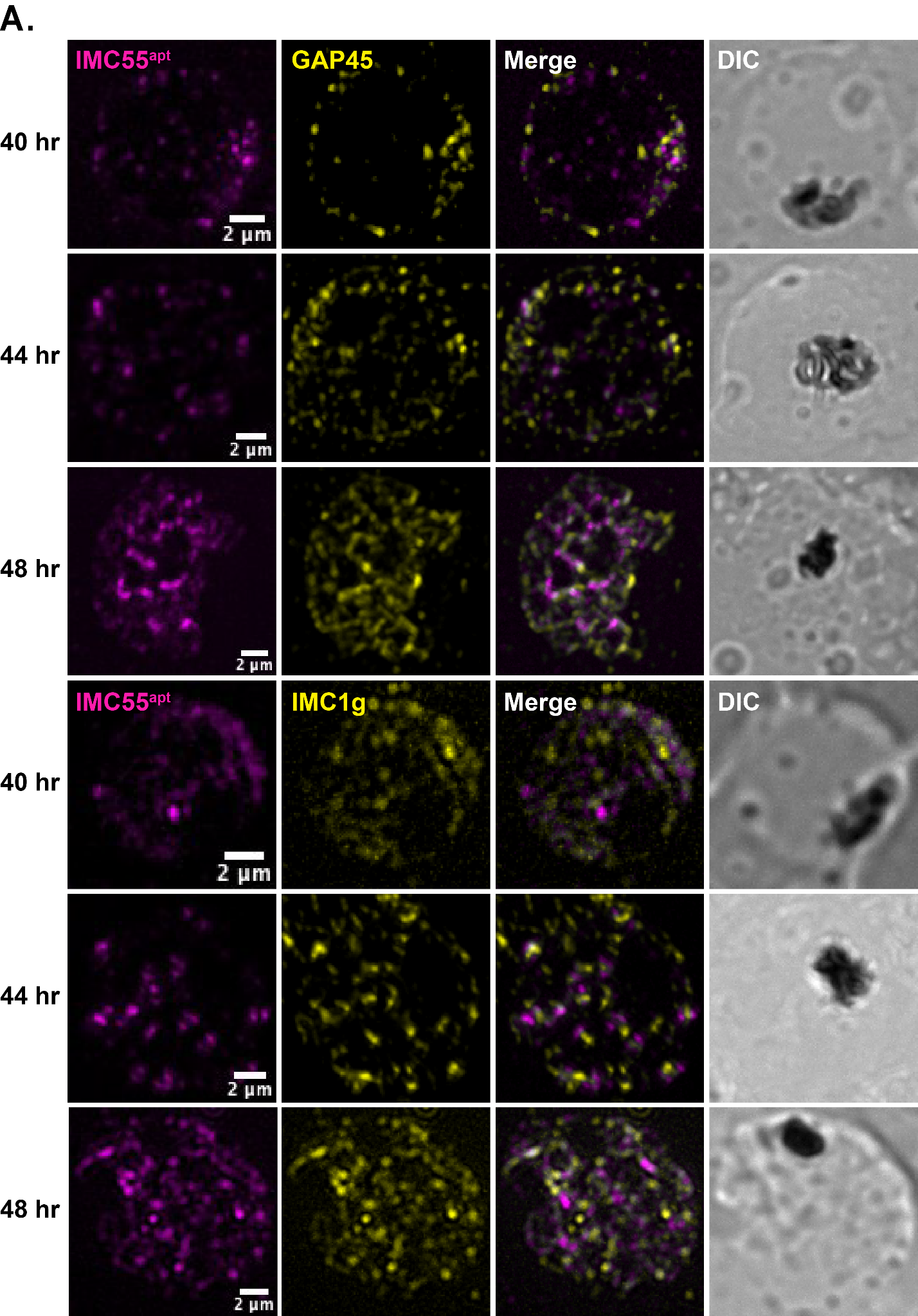


**Supplemental Figure 4. Expression of IMC55^apt^ during schizogony.** (A) Representative images from immunofluorescence assay showing the expression of IMC55^apt^ (anti-HA, in magenta) fixed during schizogony at 40-, 44-, and 48-hours post-invasion and stained with IMC markers anti-GAP45 and anti-IMC1g (in yellow). Images were collected as a Z-stack and projected as a single image. Representative images from 3 biological replicates.

**
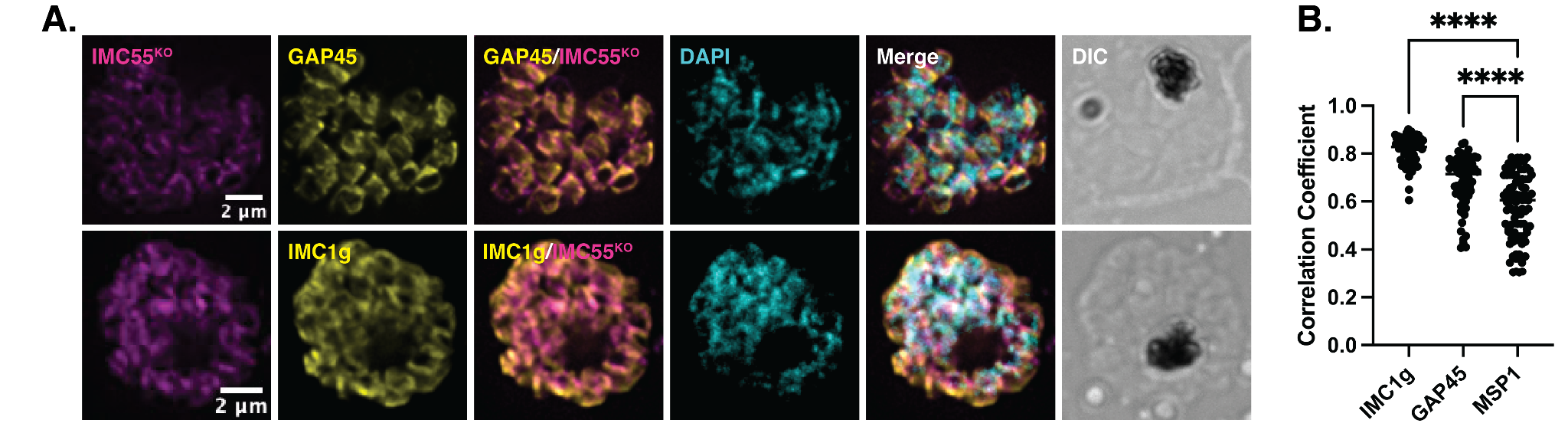
**

**Supplemental Figure 5. Colocalization of IMC55^KO^ with IMC markers.** (A) Representative images from immunofluorescence assay of IMC55^KO^ late schizonts fixed with acetone, then stained with specific antibodies. Antibodies used were anti-HA (magenta), anti-GAP45 (in yellow, top), anti-IMC1g, and the nuclear stain DAPI (cyan). Images were collected as a Z-stack, deconvolved, then projected as a single image. Representative images from 3 biological replicates. (B) Quantification of IMC55 colocalization with IMC markers and MSP1 as calculated using Pearson’s correlation coefficient (PCC). (n = 3 biological replicates, totaling 25 cells imaged with RON4 and 28 cells imaged with RAP1; Error bars = SEM; ****p < 0.0001 by unpaired two-tailed *t* test).
