# Supplementary material for: The inner membrane complex protein, IMC55, is dispensable for intraerythrocytic development of Plasmodium falciparum
Source: Microbiol Spectr. 2026 Jun 10;14(7):e03656-25. doi: 10.1128/spectrum.03656-25 (PMC13339899; doi:10.1128/spectrum.03656-25)
Supplement: Reviewer comments [file reviewer-comments.pdf]

AMERICAN SOCIETY FOR MICROBIOLOGY | Microbiology Spectrum

The inner membrane complex protein, IMC55, is dispensable for intraerythrocytic development of Plasmodium falciparum

Grace Vick, Manuel Fierro, Annabella Castiglione, Lenna Park, Carrie Brooks, and Vasant Muralidharan

Corresponding Author(s): Vasant Muralidharan, The University of Georgia

Review Timeline:

Submission Date:	November 11, 2025
Editorial Decision:	December 4, 2025
Revision Received:	April 15, 2026
Accepted:	May 6, 2026

Editor: Monica Mugnier

Reviewer(s): Disclosure of reviewer identity is with reference to reviewer comments included in decision letter(s). The following individuals involved in review of your submission have agreed to reveal their identity: Sabrina Absalon (Reviewer #2)

Transaction Report:

(Note: With the exception of the correction of typographical or spelling errors that could be a source of ambiguity, letters and reports are not edited. The original formatting of letters and referee reports may not be reflected in this compilation.)

DOI: https://doi.org/10.1128/spectrum.03656-25

Re: Spectrum03656-25 (The inner membrane complex protein, IMC55, is dispensable for intra-erythrocytic development of Plasmodium falciparum)

Dear Prof. Vasant Muralidharan:

Thank you for the privilege of reviewing your work. Below you will find my comments, instructions from the Spectrum editorial office, and the reviewer comments.

Both reviewers found the paper to be well done and useful for the field. They have suggested some minor modifications.

Please return the manuscript within 60 days; if you cannot complete the modification within this time period, please contact me. If you do not wish to modify the manuscript and prefer to submit it to another journal, notify me immediately so that the manuscript may be formally withdrawn from consideration by Spectrum.

Revision Guidelines
To submit your modified manuscript, log into the submission site at https://spectrum.msubmit.net/cgi-bin/main.plex. Go to Author Tasks and click the appropriate manuscript title to begin. The information you entered when you first submitted the paper will be displayed; update this as necessary. Note the following requirements:

• Upload point-by-point responses to the issues raised by the reviewers in a file named "Response to Reviewers," NOT in your cover letter.
• Upload a compare copy of the manuscript (without figures) as a "Marked-Up Manuscript" file.
• Upload a clean .DOC/.DOCX version of the revised manuscript and remove the previous version.
• Each figure must be uploaded as a separate, editable, high-resolution file (TIFF or EPS preferred), and any multipanel figures must be assembled into one file.
• Any supplemental material intended for posting by ASM should be uploaded with their legends separate from the main manuscript. You can combine all supplemental material into one file (preferred) or split it into a maximum of 10 files with all associated legends included.

For complete guidelines on revision requirements, see our Submission and Review Process webpage. Submission of a paper that does not conform to guidelines may delay acceptance of your manuscript.

Data availability: ASM policy requires that data be available to the public upon online posting of the article, so please verify all links to sequence records, if present, and make sure that each number retrieves the full record of the data. If a new accession number is not linked or a link is broken, provide Spectrum production staff with the correct URL for the record. If the accession numbers for new data are not publicly accessible before the expected online posting of the article, publication may be delayed; please contact production staff (Spectrum@asmusa.org) immediately with the expected release date.

Publication Fees: For information on publication fees and which article types are subject to charges, visit our website. If your manuscript is accepted for publication and any fees apply, you will be contacted separately about payment during the production process; please follow the instructions in that e-mail. Arrangements for payment must be made before your article is published.

ASM Membership: Corresponding authors may join or renew ASM membership to obtain discounts on publication fees. Need to upgrade your membership level? Please contact Customer Service at Service@asmusa.org.

The ASM Journals program strives for constant improvement in our submission and publication process. Please tell us how we can improve your experience by taking this quick Author Survey.

Thank you for submitting your paper to Spectrum.

Sincerely,
Monica Mugnier
Editor
Microbiology Spectrum

Reviewer #1 (Comments for the Author):

IMC55 Manuscript (Spectrum03656-25)

The manuscript from Vick and colleagues provides a characterization of a non-essential IMC protein, IMC55. PF3D7_0522600, named IMC55 by the authors, was initially identified as a putative interactor with PfERC. Previous work from the Gilberger lab

already demonstrated that the protein is IMC-localized, displaying a cramp like initial appearance. This localization was termed "group A" and is similar to PfGAP45 or the GAPMs. The authors corroborate that protein does localize to the IMC with some excellent microscopy, including expansion microscopy.

Genome-wide transposon screens in P. falciparum and P. knowlesi suggested that the protein was essential for asexual replication. In contrast, the ortholog had been successfully knocked out in the rodent parasite.

After initially showing that there was no phenotypic consequence for inducible knockdown of the proteins, the authors generate an inducible knockout. This also does not have a fitness defect following knockout. Thus, the authors conclude that the protein is non-essential for the asexual blood stage.

Major
1. Figure 1B seems like it is not helpful. The background gray of the different stages is not well explained. Also, the inclusion of "Conserved Pf protein" is unclear. This protein, PF3D7_1117400 is not evaluated in the study. This panel should be removed.

Minor
1. Line 72-73. The description of the IMC is always a bit difficult, especially for the non-expert reader. This description, while technically correct, is a little misleading. I suggest some clarity by stating the three bilayer membranes include the two alveoli membrane and the plasma membrane. As it is currently, it is a little ambiguous if there are 3 lipid bilayers in addition to the plasma membrane or, correctly, including the plasma membrane.
2. Some proteins are named with preceding Pf, like PfERC. Other proteins, like PMX and ATP6, do not have the preceding Pf. I think either convention is fine, but it should be consistent throughout the manuscript.
3. Line 179 - Instead of ~70%, it would be better to give a mean +/- SD percent.
4. Line 180-182. I do not think that the data demonstrate a direct role for PfERC in invasion. While this is purely semantic, it is more correct to say that "PfERC-deficient merozoites have impaired invasion". Also, the merozoites lacking PfERC are not "unable to invade" because the invasion defect is only 70%.
5. Line 192 - It is probably no longer needed to mention that CRISPR/Cas-9 was used to make transgenic parasites.
6. In methods section, references should be added for antibodies against parasite proteins.

Reviewer #2 (Comments for the Author):

Summary of the study
In this manuscript, the authors explore the inner membrane complex (IMC) multi-pass transmembrane protein "IMC55" (Pf3D7_0522600), which they identify as a potential interacting partner of the ER-resident calcium-binding protein PfERC. Using a PfERC co-IP/mass spectrometry workflow to nominate candidates, they focus on IMC55 and then assess its role during the intra-erythrocytic asexual cycle. Although previous genetic screens predicted IMC55 to be essential, the authors employ two complementary conditional strategies (TetR-aptamer knockdown and DiCre-mediated knockout) and find that IMC55 is dispensable (or functionally redundant) for asexual blood-stage replication.
General assessment
Overall, this is a well-executed and clearly organized study that employs suitable genetic tools and imaging techniques to investigate IMC55 function in blood stages. The conclusion that IMC55 is not essential for asexual replication. despite predictions from screening datasets, is valuable for the field and serves as an important reminder that predictions of essentiality need experimental validation. I have only minor suggestions to enhance clarity, framing, and presentation.

Major comments
1. Clarify scope and emphasis of the PfERC-related results (positioning of the story)
The first two Results subsections focus heavily on PfERC (interactor identification and PfERC functional data), and this emphasis was not fully apparent to me from the abstract/introduction framing. Since PfERC appears to be used both as (i) the discovery route for IMC55 and (ii) a parallel functional thread (invasion-related), I suggest revising the abstract/introduction to more explicitly preview that the paper contains substantial PfERC-centered results, not only IMC55 characterization.

Alternatively, if the PfERC invasion findings are meant to be auxiliary, consider tightening that section and making the "PfERC → IMC55 nomination" logic more straightforward so the manuscript read as a single cohesive narrative rather than two intertwined stories.
2. Timepoint wording is ambiguous (line ~209).
In the IMC55apt experiments, the text states that synchronized ring-stage parasites were cultured with or without aTc, and lysates were collected at 24/48/72 hours, then reports a reduction "by 24 hours post-aTc removal." This is probably correct, but I recommend explicitly specifying the reference point in the first sentence (e.g., "24/48/72 hours after aTc removal" vs. "hours post-invasion while maintained without aTc") to avoid confusion.
3. Unit typo for Compound 1 concentration (line ~222)
The Results text states "1.5 μm C1," which reads as a length unit rather than concentration. This should be "1.5 μM." Notably, the Methods section appears to use the correct concentration unit ("1.5 μM Compound 1"), so please ensure consistency

between Results and Methods.
4. Discussion redundancy and integration of screening predictions (P. knowlesi)
The Discussion section repeats, in some areas, the same "KD not sufficient → KO shows non-essential" narrative already clearly addressed in the Results. I recommend shortening that part and using the extra space to discuss the interesting discrepancy with published essentiality predictions. Is IMC55 also predicted to be essential in the recent published screen in P. knowlesi?
5. Stage-specific roles: expand Discussion beyond asexual blood stages
The manuscript acknowledges that IMC55 may be necessary in the mosquito or liver stages but does not thoroughly discuss sexual-stage development, despite evidence that IMC-associated proteins exhibit stage-specific essentiality (e.g., phenotypes specific to gametogenesis, such as PfBLEB; PMID: 35972967). I suggest expanding this Discussion and, if possible, including minimal supporting data or a clearer plan for future experiments.
6. IMC55 expression/localization across segmentation
The images shown are of segmented schizonts. Do the authors see IMC55 expression and "ring-like" IMC localization throughout segmentation (similar to markers like GAP45), or does the signal only appear later? Even a basic staging series by IFA (early/mid/late schizonts) would help readers understand how IMC55 fits into the overall IMC assembly timeline. (If new experiments are beyond the scope, a brief statement explaining what was or wasn't observed at different stages would still be useful.)

Minor comments / editorial issues
• Line ~62 wording: "To accomplish this feat" likely should be "To accomplish this fate," per the intended meaning.
• Formatting: Between lines ~127-136, there is increased line spacing in the PDF; please correct typesetting for readability.
• Figure quality: In the merged PDF, several figure panels (especially merged images) appear low resolution. Please provide higher-quality figure files in the revised submission (and ensure text/labels remain crisp).

Response to Reviewers

Reviewer #1 (Comments for the Author):

Major

Comment 1. Figure 1B seems like it is not helpful. The background gray of the different stages is not well explained. Also, the inclusion of "Conserved Pf protein" is unclear. This protein, PF3D7_1117400 is not evaluated in the study. This panel should be removed.

Response: We agree that this figure should be edited to improve clarity. Therefore, we have removed the background gray from the trophozoite stage and have excluded the "conserved Pf Protein (Pf3D7_1117400)" from both figure 1B and 1C since this protein is not discussed in this paper.

Minor

1. Line 72-73. The description of the IMC is always a bit difficult, especially for the non-expert reader. This description, while technically correct, is a little misleading. I suggest some clarity by stating the three bilayer membranes include the two alveoli membrane and the plasma membrane. As it is currently, it is a little ambiguous if there are 3 lipid bilayers in addition to the plasma membrane or, correctly, including the plasma membrane.

Response: To clarify the description of the IMC, we have changed the wording to be more clear. Below are the updated lines 82-87.

"The IMC is part of the three-bilayer membranous cytoskeleton which consists of the parasite plasma membrane and two membranes composed from a series of flattened vesicles termed alveoli (Morrissette & Sibley, 2002). These alveoli and interacting proteins sit juxtaposed underneath the plasma membrane and are supported by a network of filamentous proteins that make up the subpellicular network (SPN) and underlying microtubules (Mann & Beckers, 2001; Morrissette & Sibley, 2002)."

2. Some proteins are named with preceding Pf, like PfERC. Other proteins, like PMX and ATP6, do not have the preceding Pf. I think either convention is fine, but it should be consistent throughout the manuscript.

Response: We agree and we are following the naming conventions set in prior publications. We have changed ATP6 to PfATP6 and ERC to PfERC throughout the manuscript. Prior publications abbreviate plasmepsin X as PMX, apical membrane antigen 1 as AMA1 and merozoite surface protein 1 as MSP1 respectively. Hence, we are being consistent with the literature to avoid confusion.

3. Line 179 - Instead of ~70%, it would be better to give a mean +/- SD percent.

Response: Changed.

4. Line 180-182. I do not think that the data demonstrate a direct role for PfERC in invasion. While this is purely semantic, it is more correct to say that "PfERC-deficient merozoites have impaired invasion". Also, the merozoites lacking PfERC are not "unable to invade" because the invasion defect is only 70%.
Response: We agree the data does not directly demonstrate a direct role for PfERC in invasion and have thus altered our conclusion to state on line 181-183, “These data show that PfERC likely has a secondary function during invasion, and when manually released from the RBC, merozoites lacking PfERC have significantly impaired RBC invasion.”

5. Line 192 - It is probably no longer needed to mention that CRISPR/Cas-9 was used to make transgenic parasites.
Response: We have removed the text “CRISPR/Cas-9 engineering” on line 192. The text now states:
“We modified the endogenous target locus by incorporating into the repair template a Blastacidin-S (BSD) drug selection cassette, a 3X HA epitope tag, and the 10X *tetR* aptamer (Fig. 2A).”

6. In methods section, references should be added for antibodies against parasite proteins.
Response: Thank you for this comment, we have corrected the methods section to include the relevant citation where possible. The text now includes references for antibodies mouse anti-MSP1 (line 562), rabbit anti-IMC1g (line 563), rabbit anti- GAP45 (line 563).

Reviewer #2 (Comments for the Author):

Summary of the study
In this manuscript, the authors explore the inner membrane complex (IMC) multi-pass transmembrane protein "IMC55" (Pf3D7_0522600), which they identify as a potential interacting partner of the ER-resident calcium-binding protein PfERC. Using a PfERC co-IP/mass spectrometry workflow to nominate candidates, they focus on IMC55 and then assess its role during the intra-erythrocytic asexual cycle. Although previous genetic screens predicted IMC55 to be essential, the authors employ two complementary conditional strategies (TetR-aptamer knockdown and DiCre-mediated knockout) and find that IMC55 is dispensable (or functionally redundant) for asexual blood-stage replication.

General assessment

Overall, this is a well-executed and clearly organized study that employs suitable genetic tools and imaging techniques to investigate IMC55 function in blood stages. The conclusion that IMC55 is not essential for asexual replication. despite predictions from screening datasets, is valuable for the field and serves as an important reminder that predictions of essentiality need experimental validation. I have only minor suggestions to enhance clarity, framing, and presentation.

Response: We appreciate the positive assessment of our study by the reviewer. The resubmission incorporates the reviewer's suggestions to improve the clarity of the manuscript.

Major comments

1. Clarify scope and emphasis of the PfERC-related results (positioning of the story)
The first two Results subsections focus heavily on PfERC (interactor identification and PfERC functional data), and this emphasis was not fully apparent to me from the abstract/introduction framing. Since PfERC appears to be used both as (i) the discovery route for IMC55 and (ii) a parallel functional thread (invasion-related), I suggest revising the abstract/introduction to more explicitly preview that the paper contains substantial PfERC-centered results, not only IMC55 characterization.

Alternatively, if the PfERC invasion findings are meant to be auxiliary, consider tightening that section and making the "PfERC → IMC55 nomination" logic more straightforward so the manuscript read as a single cohesive narrative rather than two intertwined stories.
Response: We thank the reviewer for this excellent suggestion. We have changed the abstract as well as introduction to improve the presentation of this story. The paper now better illustrates our reasoning. We saw that PfERC had a secondary effect on parasite invasion and interaction studies identified several putative invasion ligands. While almost all of these invasion ligands are well studied, IMC55 was a novel gene without a biological function. Since the IMC clearly functions in invasion, we wanted to assess if IMC55 functioned in this process.

2. Timepoint wording is ambiguous (line ~209).
In the IMC55apt experiments, the text states that synchronized ring-stage parasites were cultured with or without aTc, and lysates were collected at 24/48/72 hours, then reports a reduction "by 24 hours post-aTc removal." This is probably correct, but I recommend explicitly specifying the reference point in the first sentence (e.g., "24/48/72 hours after aTc removal" vs. "hours post-invasion while maintained without aTc") to avoid confusion.

Response: We have edited the text to improve clarity regarding our experimental conditions when parasites are cultured without aTc. The updated text on line 208-210 now states, “To confirm that expression of IMC55 requires aTc, we cultured tightly synchronized IMC55^{apt} early ring-stage parasites with or without 500 nM aTc and collected lysates at 24, 48, and 72 hours post aTc removal.”

3. Unit typo for Compound 1 concentration (line ~222)
The Results text states "1.5 µm C1," which reads as a length unit rather than concentration. This should be "1.5 µM." Notably, the Methods section appears to use the correct concentration unit ("1.5 µM Compound 1"), so please ensure consistency between Results and Methods.
Response: The typo has been fixed to state 1.5µM C1.

4. Discussion redundancy and integration of screening predictions (P. knowlesi)
The Discussion section repeats, in some areas, the same "KD not sufficient → KO shows non-essential" narrative already clearly addressed in the Results. I recommend shortening that part and using the extra space to discuss the interesting discrepancy with published essentiality predictions. Is IMC55 also predicted to be essential in the recent published screen in P. knowlesi?
Response: We have removed some of the repetitive KD to KO text and kept a few essential discussion points here, especially that despite robust knockdown with the TetR system, there was no essentiality defect observed. However, this does not necessarily mean that it is not essential. In several other cases, even small amounts of residual protein is enough to continue asexual growth.
We have included more discussion on the P. knowlesi transposon screen. IMC55 is predicted to be essential in that screen that was completed to near saturation (lines 380-391).

5. Stage-specific roles: expand Discussion beyond asexual blood stages
The manuscript acknowledges that IMC55 may be necessary in the mosquito or liver stages but does not thoroughly discuss sexual-stage development, despite evidence that IMC-associated proteins exhibit stage-specific essentiality (e.g., phenotypes specific to gametogenesis, such as PfBLEB; PMID: 35972967). I suggest expanding this Discussion and, if possible, including minimal supporting data or a clearer plan for future experiments.
Response: This is an excellent suggestion. We have included this reference and expanded the discussion to include gametocytes as well as exo-erythrocytic stages of growth (lines 392-402).

6. IMC55 expression/localization across segmentation

The images shown are of segmented schizonts. Do the authors see IMC55 expression and "ring-like" IMC localization throughout segmentation (similar to markers like GAP45), or does the signal only appear later? Even a basic staging series by IFA (early/mid/late schizonts) would help readers understand how IMC55 fits into the overall IMC assembly timeline. (If new experiments are beyond the scope, a brief statement explaining what was or wasn't observed at different stages would still be useful.)
Response: These data are now included in Supplemental Figure 4.

Minor comments / editorial issues

• Line ~62 wording: "To accomplish this feat" likely should be "To accomplish this fate," per the intended meaning.
Response: We have edited the text to improve clarity (line 73). The text now states, "The IMC acts as a highly specialized scaffold to facilitate parasite structural integrity, cell division, and motility."

• Formatting: Between lines ~127-136, there is increased line spacing in the PDF; please correct typesetting for readability.
Response: Fixed.

• Figure quality: In the merged PDF, several figure panels (especially merged images) appear low resolution. Please provide higher-quality figure files in the revised submission (and ensure text/labels remain crisp).
Response: High-resolution TIFFs have been provided in the resubmission.

Re: Spectrum03656-25R1 (The inner membrane complex protein, IMC55, is dispensable for intraerythrocytic development of Plasmodium falciparum)

Dear Prof. Vasant Muralidharan:

Your manuscript has been accepted, and I am forwarding it to the ASM production staff for publication. Your paper will first be checked to make sure all elements meet the technical requirements. ASM staff will contact you if anything needs to be revised before copyediting and production can begin. Otherwise, you will be notified when your proofs are ready to be viewed.

Data Availability: ASM policy requires that data be available to the public upon online posting of the article, so please verify all links to sequence records, if present, and make sure that each number retrieves the full record of the data. If a new accession number is not linked or a link is broken, provide production staff with the correct URL for the record. If the accession numbers for new data are not publicly accessible before the expected online posting of the article, publication may be delayed; please contact ASM production staff immediately with the expected release date.

Publication Fees: For information on publication fees and which article types have charges, please visit our website. We have partnered with Copyright Clearance Center (CCC) to collect author charges. If fees apply to your paper, you will receive a message from no-reply@copyright.com with further instructions. For questions related to paying charges through RightsLink, please contact CCC at ASM_Support@copyright.com or toll free at +1-877-622-5543. CCC makes every attempt to respond to all emails within 24 hours.

ASM Membership: Corresponding authors may join or renew ASM membership to obtain discounts on publication fees. Need to upgrade your membership level? Please contact Customer Service at Service@asmusa.org.

Embargo Policy: A press release may be issued as soon as the manuscript is posted on the Spectrum Latest Articles webpage. The corresponding author will receive an email with the subject line "ASM Journals Author Services Notification" when the article is available online.

The ASM Journals program strives for constant improvement in our submission and publication process. Please tell us how we can improve your experience by taking this quick Author Survey.

Thank you for submitting your paper to Spectrum.

Sincerely,
Monica Mugnier
Editor
Microbiology Spectrum

Reviewer #1 (Comments for the Author):

The authors have addressed my previous concerns.

Reviewer #2 (Comments for the Author):

The authors submitted a thoughtful and robust resubmission. Congratulations to all on a beautiful story.
